# Using functional MRI neurofeedback to modulate self-blame in major depressive disorder: A pilot study

**DOI:** 10.1016/j.nicl.2026.104027

**Published:** 2026-06-22

**Authors:** Alex Nagle, Alessandro Colasanti, Roland Zahn, Chris Racey, Michael Lührs, Samira Bouyagoub, Christina Kampoureli, Jorge Moll, James Stone

**Affiliations:** a Department of Clinical Neuroscience, Brighton and Sussex Medical School, Brighton, England, United Kingdom; b Institute of Psychiatry, Psychology, and Neuroscience, King's College London, London, England, United Kingdom; c School of Psychology, University of Sussex, Brighton, England, United Kingdom; d Research Department, Brain Innovation B.V, Maastricht, Netherlands; e Department of Cognitive Neuroscience, Faculty of Psychology and Neuroscience, Maastricht University, Maastricht, Netherlands; f Clinical Imaging Sciences Centre, Brighton and Sussex Medical School, Brighton, England, United Kingdom; g Cognitive and Behavioral Neuroscience Unit, IDOR Pioneer Science Initiative, D'Or Institute for Research and Education (IDOR), Rio de Janeiro, Brazil; h Sussex Partnership NHS Foundation Trust, Worthing, England, United Kingdom

**Keywords:** fMRI neurofeedback, Depression, Self-blame, Guilt, Indignation, Subgenual cingulate cortex

## Abstract

*Background:* Growing evidence implicates self-blame-related neural networks in the pathophysiology of major depressive disorder (MDD). fMRI neurofeedback is an emerging technology with the potential to deliver interventions targeting the neural substrates of self-blame, but its feasibility and potential mechanism in current MDD remain unresolved. *Methods:* The current pilot study employed a single-session, exploratory neurofeedback trial harnessing the subgenual cingulate cortex (SCC) BOLD activity as the sole training target. Two active interventions were compared. In Intervention A (*n* = 10), participants were encouraged during neurofeedback to upregulate their SCC activity during a ‘guilt’ task and downregulate it during an ‘indignation’ task. In Intervention B (*n* = 10), participants were encouraged to do the opposite. *Results:* Clinical scores improved significantly across interventions, although no significant intervention differences were detected. Neurofeedback performance was variable across participants and conditions, with significant group-level regulation in the intended direction observed only for the indignation condition in Intervention B. A whole-brain analysis using a uniform preprocessing pipeline revealed no clusters surviving correction for multiple comparisons. *Conclusion:* The results support the feasibility of the protocol and suggest that engagement with negative autobiographical memories during SCC-oriented neurofeedback can be delivered safely in this small sample. Group-level target engagement was inconsistent, but the observation that SCC regulation was most achievable during indignation tentatively suggests this region may be more functionally heterogeneous for causal agency representations than commonly assumed. The absence of significant whole-brain effects under a uniform pipeline indicates that any neural changes were not robust at the group level in this small sample. In summary, the study provides preliminary feasibility and safety data, alongside estimates of variance, to inform more adequately powered investigations into SCC-oriented neurofeedback for depression.

## Highlights

• Clinical improvements were observed following the single-session protocol.
• SCC modulation was most achievable during indignation memory recall.
• Engaging with negative autobiographical memories was safe and well-tolerated.
• Whole-brain target-engagement effects did not survive a uniform fMRI pipeline.

## 1 Introduction

Growing evidence implicates self-blame-related neural networks in the pathophysiology of recurrent major depressive disorder (MDD) (Fennema et al., 2023; Lythe et al., 2015). Functional magnetic resonance imaging (fMRI) neurofeedback is a novel technology well-poised to deliver self-blame-specific neural interventions. During a neurofeedback session, participants are provided with information about their own local brain activity in real time, enabling them to learn how to self-modulate it for therapeutic benefit.

The subgenual cingulate cortex (SCC) and the right superior anterior temporal lobe (rSATL) have been identified from functional imaging studies as key components of a network underpinning the representation of self-blaming emotions, including their maladaptive forms in depression (Zahn et al., 2020). It is thought that the SCC (including the subgenual portion of BA24 and BA32), and the subgenual cortex (BA25) more posteriorly, represent agency and affiliative value contexts, respectively (Zahn et al., 2020), while the rSATL represents context-independent social conceptual knowledge, with integration of these sources of information being beneficial for reducing depression risk (Green et al., 2010, Green et al., 2012, Green et al., 2013). These regions have therefore been identified as promising targets for fMRI neurofeedback intervention in depression.

Only two studies to date have explicitly focussed on self-blaming biases using fMRI neurofeedback. Zahn et al. (2019) conducted a proof-of-concept randomised trial examining a single session of neurofeedback in remitted MDD. Consistent with previous research showing self-blame-selective decreases in rSATL-BA24 connectivity in remitted MDD (Green et al., 2012), participants were trained to increase rSATL-BA24 connectivity when experiencing self-blame (guilt) relative to other-blame (indignation). The active neurofeedback group successfully increased this selective connectivity and showed improvements in self-esteem compared to the control group, though effects on overall depressive symptoms were modest.

Building on this work, Jaeckle et al. (2023) compared 3 sessions of self-blame-selective fMRI neurofeedback with a self-guided, purely psychological, approach, in a randomised pilot trial of participants with current MDD. Based on previous research showing self-blame-selective increases in rSATL-BA25 connectivity predicting recurrence risk in remitted MDD (Lythe et al., 2015), the researchers reinforced patients with current MDD to decrease rSATL-BA25 connectivity during neurofeedback. Consistent with the hypothesis, participants successfully modulated brain activity as intended, and a 46% reduction in symptom severity (measured by Beck Depression Inventory-II) was observed, but there was no difference between the neurofeedback and purely psychological intervention, and the neurofeedback intervention appeared to have only benefitted non-anxious MDD participants.

Though these studies appear to successfully differentiate the functional involvement of BA24 and BA25 in depression pathophysiology (in the context of rSATL connectivity) and indicate neurofeedback protocols that complement this disparity, more recent research has questioned the assumed therapeutic directionality of these signatures (Fennema et al., 2023), particularly that of rSATL-BA25, in which higher self-blame-selective connectivity predicted a higher chance of remission in current MDD, probably reflecting a highly recurrent but more fully remitting course of MDD. This has called into question whether this is a suitable signature for fMRI neurofeedback.

The current study was designed to probe the use of the SCC (at BA24, specifically, given its closer association to self-blaming biases than BA25) as the sole neurofeedback training target to tackle self-blaming biases in current MDD. A single region-of-interest (ROI) protocol should, in theory, provide greater clarity for what constitutes a therapeutic neurofeedback paradigm in the context of self-blame and depression, before potentially reintroducing the rSATL as an accompanying target and progressing more explicitly from questions of experimental feasibility to clinical efficacy.

We tested two different SCC-oriented neurofeedback designs (Intervention A and Intervention B) in two different MDD patient groups to empirically resolve conflicting evidence regarding SCC function. Both included a self-blame (i.e. guilt) and other-blame (i.e. indignation) condition, during which participants were instructed to recall autobiographical memories evoking these emotions as a means to engage the SCC during neurofeedback. In Intervention A, participants were encouraged to increase SCC activity under the guilt condition and decrease SCC activity under indignation – a strategy based on recent evidence of self-blame-selective SCC hypo-activity in current MDD (Fennema et al., 2023). Intervention B promoted the opposite pattern, aligning with earlier findings of guilt-selective hyperactivity in remitted MDD (Fennema et al., 2021; Green et al., 2012; Lythe et al., 2020). Given that both interventions encourage SCC activity patterns that, though opposite, have been observed in healthy controls relative to depressed cohorts, we hypothesised that participants in both arms would experience clinical improvements, though we remained agnostic regarding which intervention would prove superior. To distinguish protocol-specific effects from non-specific time or placebo factors, we examined the correlation between neurofeedback regulation success and the magnitude of clinical change.

Indignation was incorporated given that it represents a reversed context of agency for blame relative to guilt (i.e. other versus self) and therefore provides a distinct theoretical contrast of agency when investigating self-blame, the condition of interest. In both interventions, participants were not given any psychological strategies to modulate their SCC activity but were predicted to learn by trial-and-error as they responded to the real-time neurofeedback.

While this investigation is designed as a pilot study to assess protocol feasibility and safety, we also conducted exploratory analyses of clinical symptoms and BOLD changes. These measures were included not to definitively establish efficacy, but to provide preliminary estimates of effect sizes and variance to inform power calculations for future definitive trials.

The primary outcome measure was a between-group comparison of the change in SCC blood‑oxygen-level-dependent (BOLD) activity from the beginning of the fMRI scan (before neurofeedback) to the end (after neurofeedback). The secondary outcome measure was the between-group comparison of the change in clinical measures (specifically, depression severity and self-esteem) from before the fMRI neurofeedback to after.

*Main hypotheses:*

1. Participants from both interventions will be able to self-modulate their SCC BOLD activity in the desired direction during neurofeedback.
2. Participants will achieve reductions in their depression score and increases in the self-esteem score following neurofeedback.

## 2 Methods

### 2.1 Ethics

This clinical proof-of-concept trial received sponsorship approval from the University of Sussex (reference code 09 STO), and Health Research Authority (HRA) ethical approval from the Brighton and Hove Research Ethics Committee (REC; reference code 22/LO/0270). The University of Sussex was the sole site for research activities. This study conformed to institutional and national standards for ethical research involving human participants, as well as with the Declaration of Helsinki. All researchers involved in the study undertook Good Clinical Practice (GCP) training. Study data have been collected, stored and retained in accordance with the Data Protection Act 2018. The study was designed, performed, and reported in accordance with the CRED-nf checklist (Ros et al., 2020) – a consensus-based set of guidelines for the experimental design and reporting of neurofeedback studies. Most ‘essential’ and ‘encouraged’ items of the checklist were met (Supplementary Materials A1).

### 2.2 Participants

Participant eligibility criteria are reported in Table 1. From 220 screened individuals, 20 (9%) completed the full study protocol. Reasons for exclusion and dropout are summarised in the CONSORT flow diagram (Supplementary Materials A2). All 20 participants successfully completed the fMRI neurofeedback intervention and subsequent assessments 2 weeks after; 17 completed the short follow-up questionnaires 4 and 6 weeks after the neurofeedback.

**Table 1:** Main participant eligibility criteria.

Inclusion Criteria	Exclusion Criteria
Aged 18 or over	Standard MRI contraindications
Access to a computer and internet connection	Uncorrectable hearing or vision deficits
Living within ∼2 h of the University of Sussex	Recreational drug use during the study, or alcohol/drug abuse in past 12 months
Diagnosis of MDD	Current daily use of pregabalin or benzodiazepines
Report of stable MDD symptoms (PHQ-2 score of >0) for ≥6 weeks before randomisation	Neurological disorders (e.g. seizures, brain injury, impaired blood flow / metabolism
	Autism spectrum or other neurodevelopmental disorders (e.g. ADHD)
	Antisocial personality disorder
	Greater than ‘low’ risk suicidality
	Current or past mania/hypomania or psychosis

Participants were allocated (double-blinded) to one of two intervention groups using minimisation to balance depression severity and gender. Each group included 10 participants (5 males in Intervention A; 3 males in Intervention B; 4 high Quick Inventory of Depressive Symptomatology (QIDS) scores in Intervention A; 5 high QIDS scores in Intervention B). Researchers were unblinded only after study completion.

### 2.3 Study visits

Visit 1 (online) included screening, collection of demographic data, and baseline clinical/psychological assessments, with the Montgomery-Asberg Depression Rating Scale (MADRS) (Williams and Kobak, 2008) and Rosenberg Self-Esteem scale (García et al., 2019) as primary clinical outcomes, and QIDS (Rush et al., 2003), Generalised Anxiety Disorder-7 (GAD-7) (Spitzer et al., 2006), and the Positive and Negative Affect Scale (PANAS) (Watson et al., 1988) as auxiliary. Visit 2 (2 weeks later) involved a single fMRI scanning session including neurofeedback. Visit 3 (another 2 weeks later) repeated the same baseline assessments. The primary clinical comparison window was from Visit 1 to Visit 3. Online follow-up questionnaires (QIDS and Rosenberg) were collected 4 and 6 weeks after the neurofeedback to monitor safety and symptom durability. All data were stored securely in encrypted case reported forms with anonymised participant codes.

## 3 fMRI paradigm

**Fig. 1:**
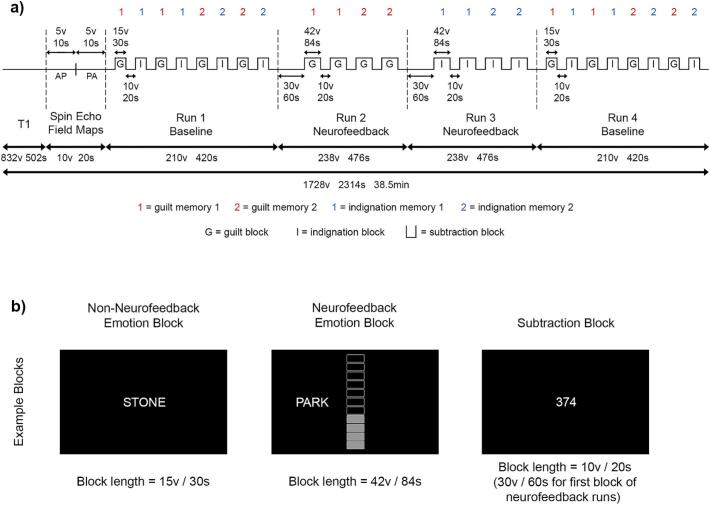
MRI paradigm design. *a) Scan Schedule. Each participant underwent just one MRI scanning session as part of the study. The main scan consisted of four runs. Before these runs, a reference anatomical image (RAI) and fieldmaps were collected. Runs 1 and 4 are baseline runs, which measure SCC BOLD activity during ‘guilt’ and ‘indignation’ tasks without neurofeedback. Runs 2 and 3 are the neurofeedback runs, where participants receive real-time visual feedback of their SCC BOLD in the form of a thermometer graphic. b) Example Visual Information. Throughout the four main experimental runs of the scan, participants viewed visual information on a screen within the scanner. In the baseline runs (Runs 1 and 4), this consisted only of a specific memory cue word during the guilt and indignation blocks, which instructed them which autobiographical memories to recall and when. The subtraction blocks within Runs 1 and 4 presented one 3-digit number on the screen, where participants had to subtract the 7 from that number sequentially in their heads. During the neurofeedback runs (Runs 2 and 3), the guilt and indignation blocks also displayed the dynamic thermometer graphic on the screen, in addition to the memory cue word. The subtraction blocks within Runs 2 and 3 were the same as in Runs 1 and 4, with just one random 3-digit number appearing on the screen at each time. The words ‘PARK’, ‘STONE’ and ‘CHAIR’ represent example memory cue words. In this figure, v = volumes and s = seconds.*

The MRI protocol consisted of four runs (Fig. 1a) A structural anatomical image and two reversed phase encoding fieldmaps were acquired first.

• Run 1 and 4 (pre- and post-neurofeedback): Each comprised 210 volumes (420 s), including 4 guilt condition and 4 indignation condition blocks (15 volumes each) alternated with 9 subtraction blocks (10 volumes each). For guilt/indignation blocks, participants recalled autobiographical memories prompted by pre-selected cue words (Fig. 1b). Subtraction blocks (representing a mental task to serially subtract 7 from a 3-digit number) provided a neutral baseline to minimise carryover of emotional and neural effects into the subsequent block.
• Run 2 and 3 (neurofeedback): Run 2 included guilt blocks, while Run 3 included indignation blocks (4 blocks of 42 volumes each). Participants recalled memories while viewing a thermometer graphic representing SCC BOLD signal. They were instructed to raise the thermometer during each block. Emotion blocks alternated with subtraction tasks (5 blocks per run). The first subtraction block (30 volumes) allowed for drift correction.

Long block lengths of 42 volumes (84 s) were chosen in order to provide sufficient time for participants to refine their strategies and achieve regulatory ‘breakthroughs’ that shorter blocks might preclude (Jaeckle et al., 2023).

Participants were not provided with any psychological strategies prior to the scan to assist them with the neurofeedback, promoting a self-guided, trial-and-error approach. The primary outcome was between-group comparison of SCC activity change from Run 1 to Run 4 (under contrasts of guilt > indignation and indignation > guilt).

### 3.1 Image acquisition

Scanning was performed on a 3 T Siemens Prisma scanner (Siemens, Erlangen, Germany) with a maximum gradient strength of 80 mT/m and a 32-channel head coil. A T1-weighted MP-RAGE sequence (0.8mm^3^ resolution, 8:22 min) was followed by collection of spin echo fieldmaps (PEPOLAR method). Functional data were acquired with T2*-weighted EPI (TR = 2000 ms, TE = 20 ms, 3.0 mm voxels, 44 slices, acceleration factor = 2, tilt angle = −30° relative to the AC-PC line).

### 3.2 ROI selection

The target region was a spherical 8 mm ROI in the anterior SCC (MNI: x = 0, y = 24, z = −8), overlapping BA24, and was selected based on prior studies linking this area to self-blaming and prosocial emotions (Krueger et al., 2007; Moll et al., 2006; Zahn et al., 2009). Coordinates used in previous self-blame-related studies (Green et al., 2012; Lythe et al., 2015; Zahn et al., 2019) vary slightly but converge on BA24 rather than BA25, given that BA25 activation itself was not shown to be associated with individual differences in self-blaming biases.

### 3.3 Neurofeedback implementation

Neurofeedback was delivered using Turbo-BrainVoyager (TBV, version 4.2.0) software (Goebel, 2012). Stimuli (cue words and thermometer display) were presented via an Expyriment-based Python script that extracted BOLD data from TBV in real time.

The thermometer display reflected percent signal change (PSC) in the SCC ROI, calculated for each volume relative to the previous volume, smoothed using a 3-volume sliding window (Supplementary Materials A4). There were 10 thermometer levels, each corresponding to a 0.1% PSC increment. For runs encouraging upregulation, the top of the thermometer therefore represented +1% relative to baseline; for downregulation runs, −1%. Feedback was delayed ∼6–7 s, reflecting the haemodynamic response and processing pipeline, and participants were informed of this before the neurofeedback session.

### 3.4 Offline preprocessing and analysis

Raw DICOMs were converted to Brain Imaging Data Structure (BIDS) format using *heudiconv* (version 1.1.6), validated, and preprocessed with *fMRIPrep* version 24.0.1 (Esteban et al., 2019). See Supplementary Materials A5 for *fMRIPrep's* full boilerplate methods text.

Due to an acquisition error, the first 7 participants had incorrect phase encoding (PE) directions and no fieldmaps (used for post-hoc image distortion correction). To avoid combining differently preprocessed images within a single group-level spatial analysis, the primary whole-brain analysis was conducted without applying fieldmap-based distortion correction to any participant (*N* = 20), ensuring a uniform preprocessing pipeline. As a secondary, exploratory analysis, fieldmap-based correction was retained for the 13 participants for whom fieldmaps were available, with the remaining 7 participants modelled by an additional third-level regressor (reported in Supplementary Materials A12). No SCC ROI voxels were lost to signal dropout in any participant. For analysis, event timing files for each task condition were created and entered into a three-level GLM in FSL FEAT:

1. First level: within-run contrasts (guilt, indignation, subtraction)
2. Second level: within-subject contrasts (Run 1, Run 4)
3. Third level: between-group contrasts (Intervention A, Intervention B)

The primary neuroimaging outcome was between-group comparison of the change in SCC BOLD activity (Run 4 > Run 1) under guilt > indignation and indignation > guilt contrasts. Secondary outcomes included between-group comparisons in clinical and psychological score changes across visits.

## 4 Results

### 4.1 Clinical analysis

When running linear mixed models (LMMs) on the clinical data, we found no significant ‘time x intervention’ interaction effect of neurofeedback on either of the primary clinical outcomes (self-esteem, as measured by the Rosenberg scale, and depression, as measured by MADRS).

**Fig. 2:**
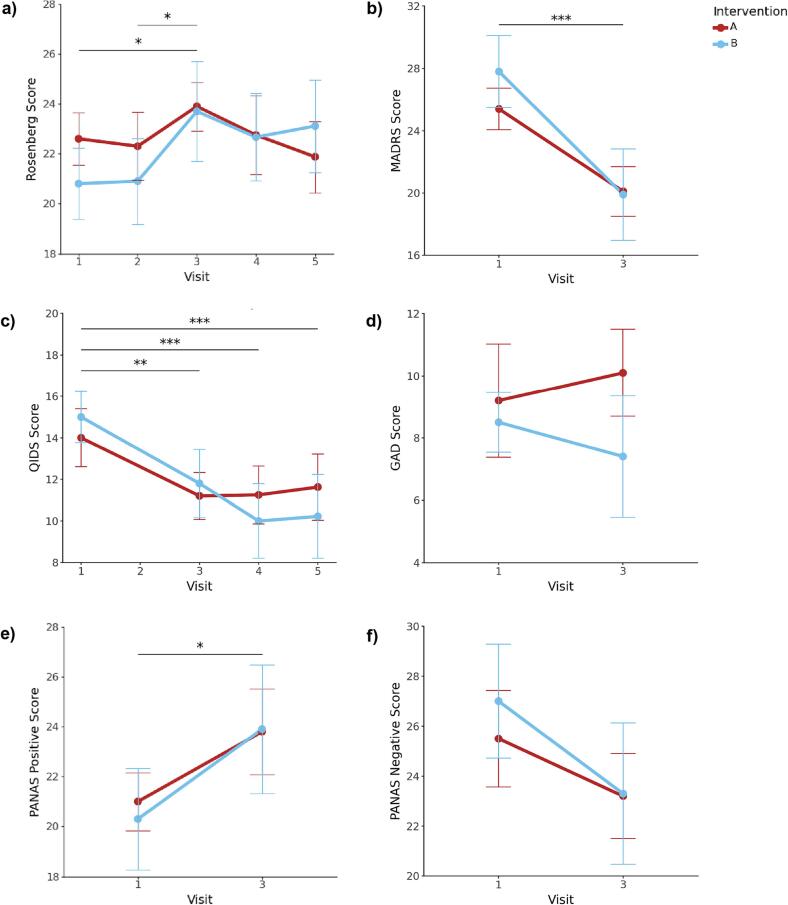
Clinical assessment scores separated by visit number and intervention. In each case, the data is first pooled by participant. a) Rosenberg self-esteem assessment scores for each intervention collected over visits 1–5. There is a significant change in score between Visit 1 and 3 (p = 0.043), and Visit 2 and 3 (p = 0.030). b) MADRS depression assessment scores for each intervention collected over visits 1 and 3. There is a significant change in score across these visits (p < 0.001). c) QIDS depression assessment scores for each intervention collected over visits 1, 3, 4, and 5. There is a significant change in score between Visit 1 and 3 (p = 0.001), Visit 1 and 4 (p < 0.001), and Visit 1 and 5 (p < 0.001). d) GAD anxiety assessment scores for each intervention collected over visits 1 and 3. e) PANAS positive assessment scores for each intervention collected over visits 1 and 3. There is a significant change in score across these visits (p = 0.014). f) PANAS negative assessment score for each intervention collected over visits 1 and 3. (*p < 0.05, **p < 0.01, ***p < 0.001). No correction for multiple comparisons was performed for these statistics.

The LMM of the Rosenberg scores (Fig. 2a) revealed a main effect of time (*p* = 0.025), with a post-hoc pairwise comparison test suggesting that this effect was driven by a significant increase in scores from Visit 1 to Visit 3 (*p* = 0.043), and from Visit 2 to Visit 3 (*p* = 0.030), representing a clinical improvement. The LMM of MADRS scores (Fig. 2b) also revealed a main effect of time (*p* < 0.001), with a significant decrease in scores between Visit 1 and 3, representing a clinical improvement.

Of the secondary clinical outcome measures, main effects of time were also reported for QIDS (*p* = 0.001; Fig. 2c) and PANAS positive affect (*p* = 0.014; Fig. 2e), once again in the therapeutic direction. LMMs of the GAD-7 (Fig. 2d) and PANAS negative affect (Fig. 2f) assessments did not return any main effects, though that of PANAS negative was close to the statistical threshold (*p* = 0.057) and represented a positive clinical improvement. Of note, multiple comparisons corrections were not applied to any of the above LMMs. Individual participant trajectories for each clinical assessment are plotted in Supplementary Materials A8.

### 4.2 Thermometer stimulus analysis

The movement of the neurofeedback thermometer stimulus by the participants represented a surrogate marker for their SCC neural activity that we could investigate independent of a GLM analysis. We explored the raw thermometer values (before they were constrained to the 10 observable levels of the thermometer), which mitigated any floor and ceiling effects in the data (Supplementary Materials A6).

We performed one-sample *t*-tests, against 0, for each of the 4 factor combinations (guilt and Intervention A, indignation and Intervention A, guilt and Intervention B, indignation and Intervention B) for each participant, to test whether participants could successfully move the thermometer in the desired direction. The results are summarised in Table 2, showing, in accordance with Supplementary Materials A6, that the expanded thermometer levels for the indignation condition in Intervention B were significantly different to 0 (more specifically, they were higher; *p* = 0.028), while the other factor combinations did not pass the significance threshold. Individual participant-level results of these tests are reported in Supplementary Materials A9, and individual participant mean expanded thermometer levels for each condition are plotted in Supplementary Materials A10.

**Table 2:** Results of one-sample t-tests of expanded thermometer levels for each factor combination against 0. (*p < 0.05, **p < 0.01, ***p < 0.001).

Condition	Intervention	Mean	Standard Deviation	Test Type	T- / W-stat	*P*-Value	Significance Level
Guilt	A	−1.11	2.28	Parametric	−1.55	0.157	–
Indignation	A	0.59	4.36	Parametric	0.43	0.678	–
Guilt	B	−0.63	2.45	Non-parametric	22	0.604	–
Indignation	B	2.09	2.51	Parametric	2.63	0.028	*

We also performed exploratory correlation analyses to investigate the relationship between the mean raw thermometer values and the change in score between Visit 1 and Visit 3 for each clinical assessment (Supplementary Materials A7). To control for the high risk of false positives across the 24 statistical tests, a Benjamini-Hochberg False Discovery Rate (FDR) correction was applied. While uncorrected analysis indicated a strong correlation between thermometer upregulation and anxiety reduction (GAD-7) in the Intervention A group (*r* = 0.77, uncorrected p = 0.01), this result did not survive FDR correction (corrected *p* > 0.05). Consequently, no statistically significant associations were confirmed. However, visual inspection reveals exploratory trends suggesting a positive relationship between thermometer movement success and clinical improvements, given that coefficient values across factor combinations tended to be positive for assessments where higher scores reflect the therapeutic direction, and negative for assessments where lower scores reflect the therapeutic direction.

### 4.3 Neuroimaging analysis

The primary whole-brain analysis used a uniform preprocessing pipeline in which fieldmap-based distortion correction was not applied to any participant (N = 20), thereby avoiding the introduction of pipeline heterogeneity into the group-level spatial analysis (Supplementary Materials A11). For the ‘indignation > guilt’ with ‘Run 4 > Run 1’ and ‘Intervention B > Intervention A' contrast, no clusters survived whole-brain cluster-corrected thresholding (cluster-forming threshold z > 2.3; cluster significance *p* < 0.05, corrected). The strongest sub-threshold signal was located in the left temporoparietal region (peak MNI coordinates −58, 6, −6; z-max = 3.64). The whole-brain analysis therefore did not provide statistically significant evidence of a between-group difference in the change in task-related activation following neurofeedback.

For completeness, an exploratory analysis was also conducted that retained fieldmap-based distortion correction for the 13 participants for whom fieldmaps were available and modelled the remaining 7 participants with an additional third-level regressor (Supplementary Materials A12). Under this mixed pipeline, two left-lateralised clusters (temporoparietal and occipital) survived cluster correction for the same contrast. However, because this approach combines images preprocessed with and without distortion correction within a single group-level spatial analysis, it is susceptible to alignment confounds in susceptibility-prone regions, and the clusters did not reproduce under the uniform pipeline reported above. This exploratory result is therefore reported in the supplementary materials only and is interpreted with caution.

## 5 Discussion

### 5.1 Summary

This double-blinded, single-session fMRI neurofeedback pilot study targeted the understudied symptom domain of self-blame in current MDD. We investigated whether patients could learn to modulate their SCC activity in real-time, and whether successful modulation shows potential therapeutic value.

The study design incorporated two active intervention arms to directly compare competing therapeutic approaches suggested by the literature. In Intervention A, participants were encouraged to upregulate their SCC BOLD activity during guilt recall and downregulate it during indignation recall, aiming to enhance the differentiation between self- and other-blaming emotion signatures as observed in healthy controls (Fennema et al., 2023). Conversely, Intervention B promoted the opposite pattern – downregulation during guilt and upregulation during indignation. This approach sought to reduce the guilt-selective SCC hyperactivity previously observed in remitted MDD, and to enhance the differentiation pattern between self- and other-blaming emotion signatures observed in other healthy controls (Fennema et al., 2021; Green et al., 2012; Lythe et al., 2020). This design allowed us to explore a fundamental uncertainty in the field regarding the optimal training direction for SCC-based neurofeedback in depression.

The study broadly met the criteria of the consensus-based CRED-nf checklist for neurofeedback study design and reporting.

### 5.2 Clinical data

Clinical results showed encouraging improvements across multiple instruments, including the primary outcomes of self-esteem (Rosenberg) and depressive symptoms (MADRS), as well as secondary measures (QIDS, PANAS positive affect), with all changes in the therapeutic direction. However, given the lack of a (sham or yoked neurofeedback) control group, these clinical improvements cannot be causally attributed to the specific modulation of SCC activity. It is possible that non-specific factors, such as emotional exposure (engaging with autobiographical memories), the supportive research environment, or placebo effects, contributed to the observed symptom reduction.

These clinical improvements align with findings from previous self-blame-related fMRI neurofeedback studies (Jaeckle et al., 2023; Zahn et al., 2019) and support the premise that direct engagement with negative emotional content – a reliable condition for modulating blame-selective network dynamics – can be conducted safely. Crucially, despite the absence of a formal mood repair procedure, the intervention was well-tolerated, with no participants exhibiting symptom exacerbation immediately post-scan.

No statistically significant clinical differences emerged between interventions, though numerical trends consistently favoured Intervention B. Despite beginning with slightly more severe symptomatology, Intervention B participants achieved comparable or superior outcomes by Visit 3. These observations, though non-significant in our small sample, provide preliminary evidence that encouraging SCC downregulation during guilt and upregulation during indignation may be more therapeutically beneficial.

### 5.3 Thermometer analysis

Analysis of the thermometer data, representing neurofeedback performance and the underlying SCC neural activity, revealed differential success rates across conditions and interventions. Most notably, only participants in Intervention B during the indignation condition demonstrated a statistically significant ability to move the thermometer in the desired direction (upward, representing SCC upregulation in this case). This finding was driven by 7 out of the 10 participants in this group successfully modulating their SCC activity above the dynamic baseline level. In contrast, Intervention A participants showed limited success during the indignation condition, and the guilt condition for both interventions reflected even poorer performance.

These performance patterns challenge our initial assumptions that guilt-related SCC modulation would prove more achievable than indignation-related modulation. The superior performance during indignation neurofeedback (particularly when an increase in SCC BOLD is being reinforced) may reflect several factors. First, the SCC's functional heterogeneity may include substantial territory devoted to processing other-blaming emotions, contrary to traditional assumptions of self-blame specificity (e.g. Green et al., 2012). Second, for depressed individuals who may possess entrenched patterns of self-blame, actively engaging with other-directed blame through indignation memories might provide a more accessible route to SCC modulation. Third, it is possible that the act of increasing activation (as required for indignation in Intervention B) is inherently easier than decreasing activation in a region already showing altered baseline activity in depression.

Interestingly, among the 7 Intervention B participants who successfully increased the thermometer during indignation (corresponding to an increase in SCC activity), 4 of them found that engaging in the indignation emotion was most effective, and 2 found that attempting to rationalise the other person's behaviour and feel compassion for them was most effective, while 1 found that a combination of these methods worked. These results provide preliminary indications that leaning into the indignation emotion associated with the memory has the most utility for increasing the level of the thermometer (when increases in SCC are being encouraged).

These performance data provide only qualified evidence of target engagement. Significant group-level regulation in the intended direction was observed in only one of the four factor combinations (the indignation condition in Intervention B), and even there was driven by 7 of the 10 participants, with the remainder showing little or no regulation. At the individual level, the direction and magnitude of thermometer movement varied substantially across participants and conditions (Supplementary Materials A9, A10), and a number of participants moved the thermometer opposite to the encouraged direction. This heterogeneity indicates that volitional access to the target signal was inconsistent across the sample and that reliable, uniform target engagement was not achieved. The neurofeedback loop was nonetheless technically feasible, and a subset of patients could modulate the feedback signal, supporting the feasibility of the protocol while underscoring the degree of variability that an adequately powered future trial would need to address.

### 5.4 Neuroimaging data

The primary uniform-pipeline whole-brain analysis did not identify any significant clusters. In the exploratory mixed-pipeline analysis (Supplementary Materials A12), two clusters of activation were observed, associated with the left temporoparietal region and the left occipital cortex; because this result did not reproduce under the uniform pipeline, the regional interpretation that follows is necessarily tentative. While these clusters did not encompass our a priori SCC ROI, the results implicate brain areas relevant to emotional processing and social cognition. The left temporoparietal cluster that was revealed explicitly involved the insular cortex, which has been recognised previously as a contributor to the self-blame network (Fennema et al., 2024). Additionally, it is possible that this cluster also implicates the temporoparietal junction and the ATL – two regions with well-established roles in the self-blame network (Lambon Ralph et al., 2010; Lythe et al., 2015; Moll et al., 2005; Zahn et al., 2009, Zahn et al., 2014).

There is a partial discrepancy between the group-level GLM results, which revealed no significant clusters under the primary uniform pipeline, and the real-time neurofeedback performance data, in which a subset of Intervention B participants upregulated SCC activity under the indignation condition. Several methodological factors may contribute to this: the thermometer tracks performance in subject-specific space, whereas the GLM requires precise spatial convergence across the group in standardised space. Given the known anatomical variability (Palomero-Gallagher et al., 2015) and susceptibility to signal dropout (Volz et al., 2019) in the subgenual cingulate, individual modulation that is genuine in subject space may nonetheless fail to converge spatially across the group. A null group-level result therefore does not by itself establish that no regulation occurred; equally, given the inconsistent thermometer performance described above, it cannot be taken as evidence that the target was reliably engaged. The whole-brain data are most appropriately read as inconclusive at the group level in this small, heterogeneous sample, rather than as evidence for the engagement of a distributed network beyond the SCC.

The occipital cluster observed in the exploratory analysis (Supplementary Materials A12) likely reflects heightened visual attention to the thermometer graphic. However, this cluster emerged specifically from the indignation > guilt contrast, and the visual characteristics of the stimulus were identical across conditions. This suggests that a factor specific to the indignation condition in Intervention B – potentially more intense monitoring of the feedback signal during blocks of greater regulatory success – may have driven the increased occipital activation, though this remains exploratory and to be tested.

Lastly, the lateralisation of effects to the left hemisphere contrasts with traditional associations of right hemisphere dominance for the processing of social cues and blaming emotions (Skipper et al., 2011; Zahn et al., 2007). It is possible that this pattern may reflect the specific cognitive demands of the neurofeedback task, which required deliberate, conscious manipulation of emotional states, rather than passive emotional experience – phenomena with putatively closer associations to the left hemisphere over the right (Brésard and Bresson, 1987; McGilchrist, 2019).

Taken together, the neuroimaging data should be regarded as inconclusive rather than positive. The primary, uniformly preprocessed analysis revealed no significant whole-brain effects, and the two clusters seen in the exploratory mixed-pipeline analysis did not reproduce under it. The broad spatial correspondence between the two analyses provides some reassurance that the exploratory clusters are not purely artefacts of differential fieldmap correction across participants; however, their failure to survive a homogeneous pipeline means they cannot be interpreted as robust evidence of regional engagement. Reduced brain coverage and increased variance from uncorrected EPI distortions in susceptibility-affected regions likely limited sensitivity, but the available data do not allow us to distinguish a genuine effect obscured by noise from an absence of effect. Any engagement of these regions during self-blame-related neurofeedback therefore remains to be established in a larger sample acquired with uniform, fieldmap-supported parameters.

These findings should also be situated within the broader and rapidly developing literature on fMRI neurofeedback for depression. Several studies targeting other regions and networks have reported more consistent regulation than was achieved here. For example, amygdala-based neurofeedback aimed at increasing responses to positive autobiographical memories has shown reproducible upregulation and associated symptom improvement in small samples (Young et al., 2014, Young et al., 2017; Zotev et al., 2011), and studies targeting frontal asymmetry or salience- and affect-related networks have likewise reported learnable control (Hamilton et al., 2016; Linhartová et al., 2019; Mehler et al., 2018; Misaki et al., 2020; Quevedo et al., 2020). By contrast, the SCC is a small, ventrally located, susceptibility-prone region in which reliable real-time signal extraction and cross-subject spatial convergence are intrinsically more difficult. The inconsistent target engagement and null whole-brain result observed here may therefore reflect, in part, the difficulty of using a single deep midline region of interest as a neurofeedback target, rather than an absence of trainable signal per se. This contrast underscores the importance of target selection – and potentially of network- or connectivity-based targets – for future self-blame-oriented neurofeedback.

### 5.5 Limitations

One principal limitation concerns the modest sample size of 20 participants, which constrained statistical power, particularly for detecting intervention differences in the current study. With only 10 participants per intervention group, even one or two outliers could substantially influence group means and apparent trends. This is particularly relevant for the clinical outcome data, where the observed numerical advantages for Intervention B, while consistent across measures, remained far from statistical significance. Whether these trends represent true effects or random variation cannot be determined without replication in larger samples, although reports suggest non-specific placebo-related effects are likely to be small for neurofeedback (Xia et al., 2024).

Many prospective participants did not meet our eligibility criteria for the study, contributing to the small sample size attained. It is possible that our criteria were too strict, particularly those related to recent drug and alcohol abuse, and symptom stability (Supplementary Materials A2). Future studies may benefit from a more inclusive recruitment strategy to ensure satisfactory samples sizes are met.

The lack of a proper control group represents a limitation for interpreting the clinical improvements. While the comparison of two active interventions addressed our primary research question concerning optimal training directions, it precluded strong conclusions about the specific efficacy of neurofeedback vs. non-specific factors.

The imbalance of comorbidities between groups represents a further limitation. Intervention B contained four participants with co-morbid PTSD, whereas Intervention A contained none (Supplementary Materials A3). Since indignation and externalised blame are central features of PTSD, the superior indignation performance in Intervention B may have been influenced by this phenotype. However, neurofeedback performance was measured as change relative to a dynamic baseline, so higher trait indignation would not necessarily facilitate – and might hinder via ceiling effects – the required upregulation. Pre-scan subjective intensity ratings of indignation memories did not differ significantly between groups (Intervention A: Mean = 6.4, SD = 0.74; Intervention B: Mean = 6.1, SD = 0.74; *t* = 0.91, *p* = 0.38). Nonetheless, future studies must control for such comorbidities.

A further limitation concerns the fMRI acquisition itself. Owing to an acquisition error, fieldmaps were available for only 13 of the 20 participants. To avoid introducing pipeline heterogeneity into the group-level spatial analysis, the primary whole-brain analysis was therefore conducted without fieldmap-based distortion correction for any participant, ensuring a uniform pipeline. This came at the cost of reduced brain coverage and increased variance from uncorrected susceptibility-induced EPI distortions, which lowered BOLD sensitivity in ventral and susceptibility-prone regions and is likely to have reduced power to detect group-level effects. The exploratory analysis that retained fieldmap correction where available, modelling its absence with a third-level regressor (Supplementary Materials A12), is reported for transparency but is itself limited by the mixing of differently preprocessed images within a single spatial analysis. A definitive test of the neuroimaging hypotheses will require a larger sample acquired with complete and uniform fieldmap data.

### 5.6 Conclusion

In summary, this pilot study supports two principal conclusions and qualifies a third. First, the single-session protocol was feasible and safe: clinical scores improved across both interventions and engaging with negative autobiographical memories during SCC-oriented neurofeedback was well tolerated, although the absence of a control group precludes any claim of efficacy. Second, target engagement was inconsistent: significant group-level regulation was confined to the indignation condition in Intervention B, individual thermometer performance was highly variable, and the primary whole-brain analysis revealed no significant effects. Third, the tentative observation that SCC regulation was most achievable during indignation, if replicated, would be consistent with greater functional heterogeneity of this region for causal-agency representations than is commonly assumed; however, given the variability of engagement and the null whole-brain result, this remains a hypothesis to be tested rather than a finding.

Given these limitations, the findings should be interpreted as hypothesis-generating. Nonetheless, the protocol was feasible and safe, a subset of participants could modulate the feedback signal, and clinical improvements were observed across interventions, providing justification for larger, adequately powered and methodologically uniform trials to refine training paradigms and address questions of target engagement and clinical efficacy.

## CRediT authorship contribution statement

**Alex Nagle:** Writing – review & editing, Writing – original draft, Visualization, Project administration, Methodology, Investigation, Formal analysis, Data curation, Conceptualization. **Alessandro Colasanti:** Writing – review & editing, Supervision, Project administration, Methodology, Funding acquisition, Conceptualization. **Roland Zahn:** Writing – review & editing, Supervision, Resources, Methodology, Funding acquisition, Conceptualization. **Chris Racey:** Writing – review & editing, Software, Resources, Formal analysis. **MichaelLührs:** Writing – review & editing, Software, Resources, Methodology. **Samira Bouyagoub:** Writing – review & editing, Software, Resources, Data curation. **Christina Kampoureli:** Resources, Formal analysis. **Jorge Moll:** Writing – review & editing, Conceptualization. **James Stone:** Writing – review & editing, Supervision, Resources, Project administration, Methodology, Funding acquisition, Conceptualization.

## Funding sources

This project constituted a PhD studentship funded by Brighton and Sussex Medical School.

## Declaration of competing interest

The authors declare the following financial interests/personal relationships which may be considered as potential competing interests: Roland Zahn reports a relationship with London Depression Institute that includes: employment. Roland Zahn reports a relationship with EMOTRA that includes:. Roland Zahn reports a relationship with Depsee Ltd. that includes:. Roland Zahn reports a relationship with Lundbeck that includes:. Roland Zahn reports a relationship with Janssen Pharmaceuticals Inc. that includes:. Roland Zahn reports a relationship with Neuraxpharm that includes:. Roland Zahn reports a relationship with D'Or Institute for Research and Education that includes:. Roland Zahn reports a relationship with Scients Institute that includes:. Co-author is a co-investigator on a Livanova-funded observational study of Vagus Nerve Stimulation for Depression (R.Z.). If there are other authors, they declare that they have no known competing financial interests or personal relationships that could have appeared to influence the work reported in this paper.

## Data Availability

Data will be made available on request.

## Appendix A Supplementary data

Supplementary material Supporting data, secondary analyses, and additional context.
