# Supplementary material for: Using functional MRI neurofeedback to modulate self-blame in major depressive disorder: A pilot study
Source: Neuroimage Clin. 2026 Jun 22;51:104027. doi: 10.1016/j.nicl.2026.104027 (PMC13319699; doi:10.1016/j.nicl.2026.104027)
Supplement: Supplementary material — Supporting data, secondary analyses, and additional context. [file mmc1.docx]

**Using functional MRI neurofeedback to modulate self-blame in major depressive disorder.**

**Appendix A: Supplementary Materials.**


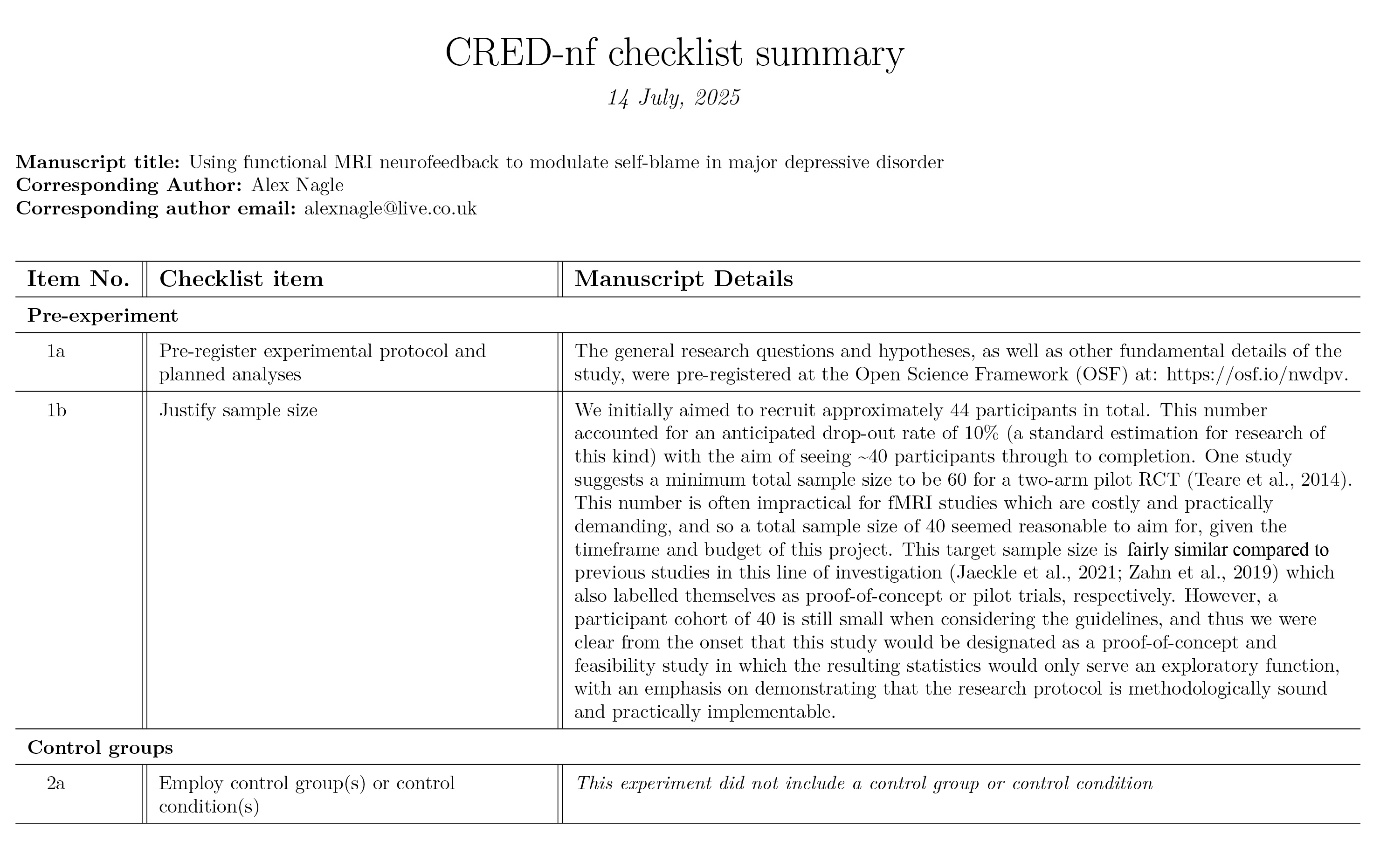

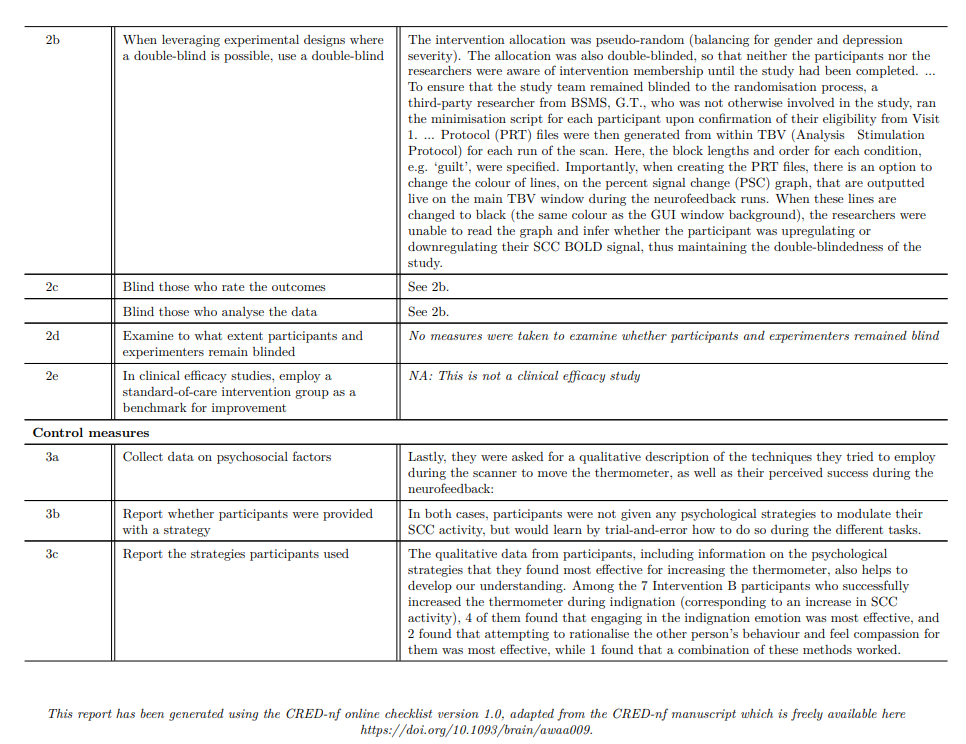
**A1 CRED-nf Checklist.**


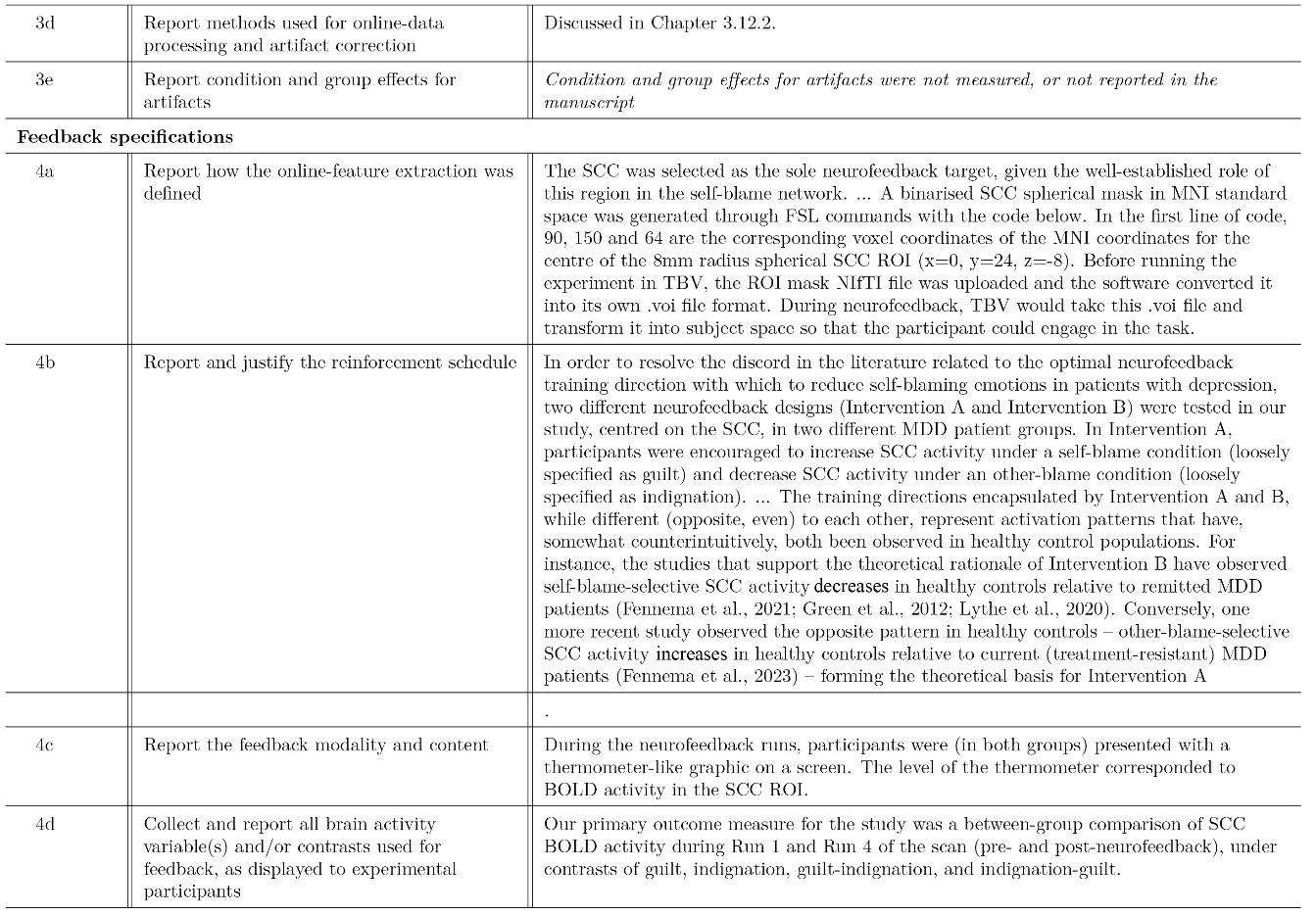

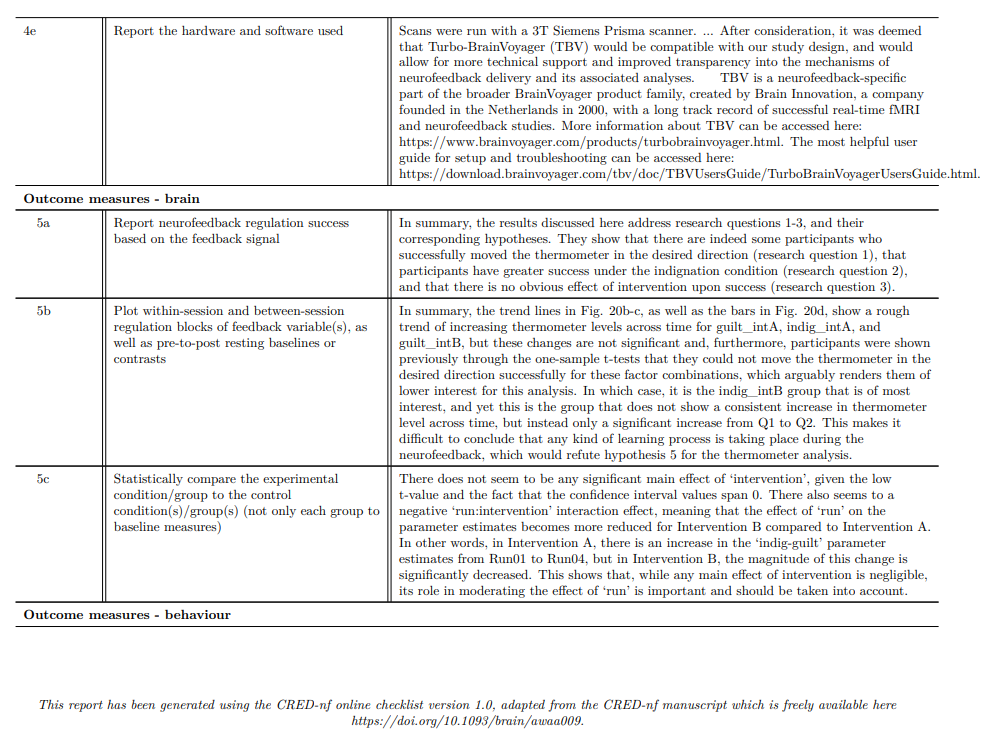


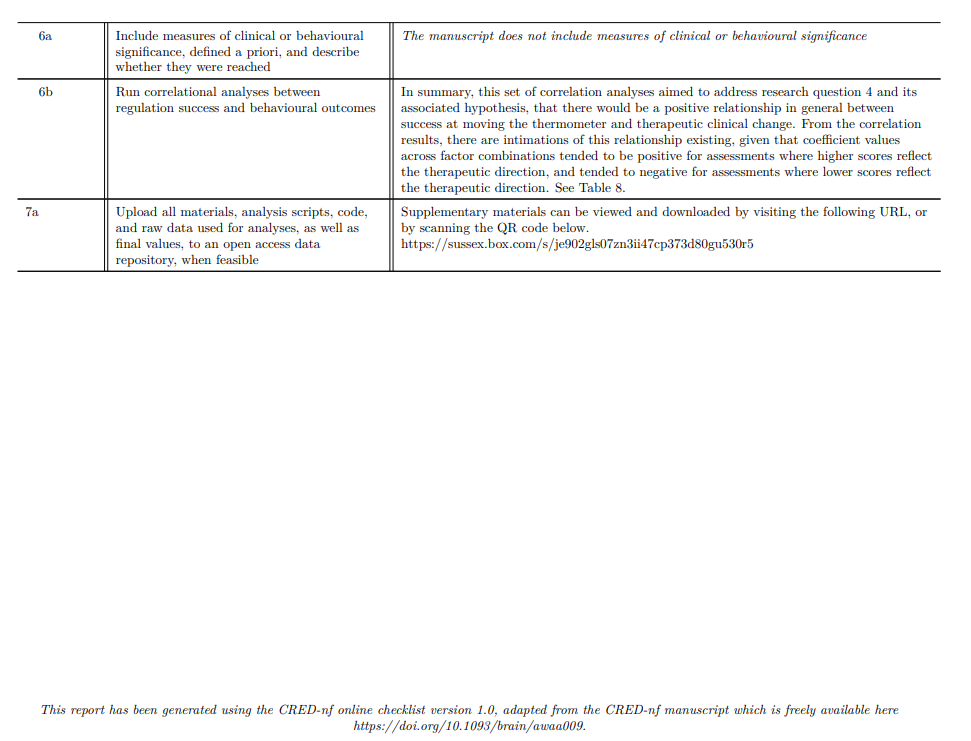


***Figure 1 CRED-nf Checklist.***

**A2 CONSORT Flow Diagram.**


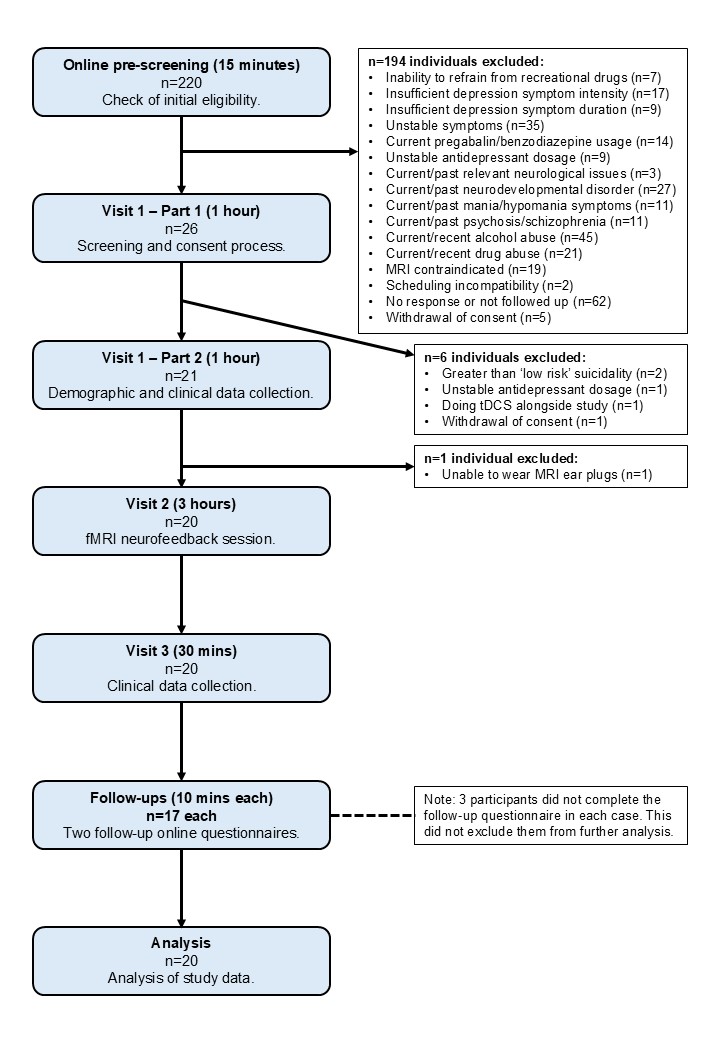


Figure 2. CONSORT flow diagram of participant recruitment and retention. *In Part 1 of Visit 1,, participants are screened thoroughly to check all eligibility criteria have been satisfied. In Part 2 of Visit 1, initial data collection takes place and a series of clinical assessments are delivered. Visit 2 consists of the MRI neurofeedback session. In Visit 3, participants undergo another set of clinical assessments as a point of comparison for observations from Visit 1. In the 2^nd^ and 4^th^ weeks following Visit 3, participants are sent two short online questionnaires related to self-esteem and depression symptoms that are used to investigate longer-term effects The reasons for participant exclusion or discontinuation from the study are listed for each stage of the diagram.*

**A3 Demographic and Clinical Features of Interventions.**

***Table 1. Summary of demographic and clinical features by intervention group.***

|  | Intervention A (n = 10) | Intervention B (n = 10) |
| --- | --- | --- |
| Average age (years) | 35.5 | 33.8 |
| Gender | 5 Male (50%), 5 Female (50%) | 3 Male (30%), 7 Female (70%) |
| Highest level of education | 1 Vocational or Similar; 4 Some University; 3 University Bachelors Degree; 2 Graduate or Professional Degree | 1 Some Secondary; 1 Completed Secondary; 3 Some University; 3 University Bachelors Degree; 2 Graduate or Professional Degree |
| Age of initial symptom onset (mean years) | 18.7 | 13.6 |
| Number of antidepressants tried (mean) | 0.7 | 0.7 |
| QIDS score (mean) | 14.0 | 15.0 |
| MADRS score (mean) | 25.4 | 27.8 |
| MSM score of treatment resistance (mean) | 5.9 | 6.1 |
| Panic disorder (Yes) | 0 / 10 (0%) | 2 / 10 (20%) |
| Agoraphobia (Yes) | 0 / 10 (0%) | 1 / 10 (10%) |
| Social anxiety disorder (Yes) | 3 / 10 (30%) | 4 / 10 (40%) |
| OCD (Yes) | 1 / 10 (10%) | 1 / 10 (10%) |
| PTSD (Yes) | 0 / 10 (0%) | 4 / 10 (40%) |
| Generalised anxiety disorder (Yes) | 4 / 10 (40%) | 1 / 10 (10%) |
| Participants with ≥1 anxiety disorder | 5 / 10 (50%) | 7 / 10 (70%) |
| Anxiety is comorbid vs anxious depression (Yes) *(denominator: those with ≥1 anxiety disorder)* | 3 / 5 (60%) | 3 / 7 (43%) |
| Rosenberg self-esteem score (mean) | 22.6 | 18.8 |
| PSI-II sociotropy score (mean) | 91.9 | 100.3 |
| PSI-II autonomy score (mean) | 73.0 | 77.2 |

**A4 BOLD PSC Calculation.**


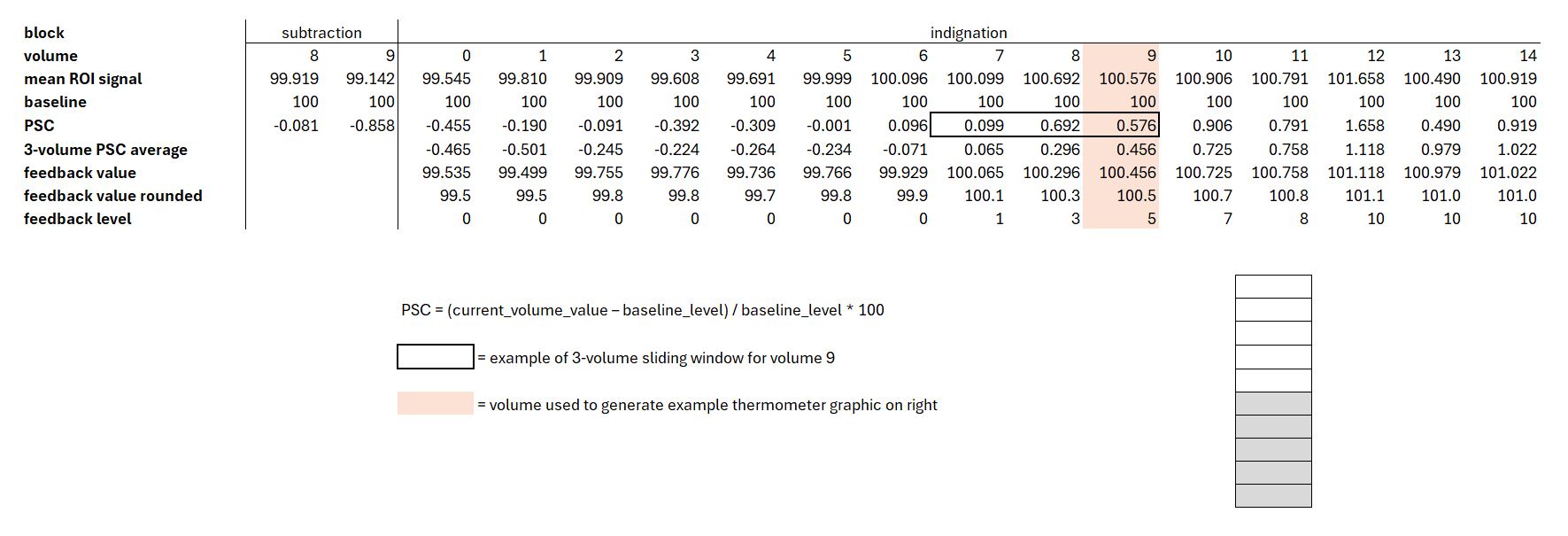


Figure 3. Example subset of BOLD data illustrating the thermometer level calculation. *The data here are derived from the last two volumes of a subtraction block, and the first 15 volumes of the subsequent indignation block from Run 3 of a participant from Intervention B, where upregulation of SCC activity is encouraged for the indignation condition. Following volume 9 on the figure as an example, one can note the PSC being calculated from the mean ROI signal and the baseline value, before being averaged across the PSC values from the previous two volumes (‘3-volume sliding window’). The resulting value is then added to the baseline value and rounded to one decimal place. The first decimal number then corresponds to the level of the thermometer shown to the participant for this volume of the run. The example thermometer for volume 9 on this figure is shown on the right, with 5 levels being filled, indicative of a 0.5% increase of the ROI BOLD signal relative to the previous volume, with the 3-volume sliding window also applied.*

**A5 fMRIPrep Boilerplate Methods Text.**

The raw DICOM images from the scanning sessions were converted into standard BIDS format, as NIfTIs. This was done using heudiconv (version 1.1.6). All participants’ BIDS-converted data were uploaded to an online ‘BIDS Validator’ tool (<https://bids-standard.github.io/bids-validator/>) and passed the checks. Next, the JSON sidecar files of the fieldmaps were edited to add an ‘IntendedFor’ key, listing the functional images that the fieldmaps should apply to for that participant.

At this point, the data was ready to undergo preprocessing via fMRIPrep (version 24.0.1), a preprocessing pipeline application for fMRI data (Esteban et al., 2019; Markiewicz et al., 2024; *SciCrunch | Research Resource Resolver*, n.d.-a), which is based on Nipype 1.8.6 (Esteban et al., 2022; Gorgolewski et al., 2011; *SciCrunch | Research Resource Resolver*, n.d.-b). The application was run within a singularity containerisation platform on a high-performance computing (HPC) cluster.

Preprocessing of B0 inhomogeneity mappings: a total of 1 fieldmap pair was found available within the input BIDS structure for 13 of the 20 participants preprocessed. B B0-nonuniformity map (or fieldmap) was estimated based on two (or more) EPI references with topup (Andersson et al., 2003; Smith et al., 2004).

Anatomical data preprocessing: a total of 1 T1-weighted (T1w) mages were found within the input BIDS dataset for each participant. The T1w image was corrected for intensity non-uniformity (INU) with N4BiasFieldCorrection (Tustison et al., 2010), distributed with ANTs 2.5.1 (Avants et al., 2008; *SciCrunch | Research Resource Resolver*, n.d.-c), and used as T1w-reference throughout the workflow. The T1w-reference was then skull-stripped with a Nipype implementation of the antsBrainExtraction.sh workflow (from ANTs), using OASIS30ANTs as target template. Brain tissue segmentation of CSF, WM, and GM was performed on the brain-extracted T1w using FAST (*SciCrunch | Research Resource Resolver*, n.d.-d; Zhang et al., 2001). Brain surfaces were reconstructed using recon-all from FreeSurfer 7.3.2 (Dale et al., 1999; *SciCrunch | Research Resource Resolver*, n.d.-e), and the brin mask estimated previously was refined with a custom variation of the method to reconcile ANTs-derived and FreeSurfer-derived segmentations of the cortical GM of Mindboggle (Klein et al., 2017). Volume-based spatial normalisation to two standard spaces (MNI152NLin2009cAsym, MNI152NLin6Asym) was performed through nonlinear registration with antsRegistration (ANTs 2.5.1), using brain-extracted version of both T1w reference and the T1w template. The following templates were selected for spatial normalisation and accessed with Template Flow 24.2.0 (Ciric et al., 2022): ICBM 152 Nonlinear Asymmetrical template version 2009c (Fonov et al., 2009; *SciCrunch | Research Resource Resolver*, n.d.-f), TemplateFlow ID MNI152NLin2009cAsym; FSL’s MNI ICBM 152 non-linear 6^th^ Generation Asymmetric Average Brain Stereotaxic Registration Model (Evans et al., 2012; *SciCrunch | Research Resource Resolver*, n.d.-d), TemplateFlow ID MNI152NLin6Asym. Grayordinate “dscalar” files containing 91k samples were resampled onto fsLR using the Connectome Workbench (Glasser et al., 2013).

Functional data preprocessing: for each of the 4 BOLD runs found per subject (across all tasks and sessions), the following preprocessing was performed. First, a reference volume was generated, using a custom methodology of fMRIPrep, for use in head motion correction. Head-motion parameters with respect to the BOLD reference (transformation matrices, and six corresponding rotation and translation parameters) are estimated before any spatiotemporal filtering using MCFLIRT (Jenkinson et al., 2002). The estimated fieldmap was then aligned with rigid-registration to the target EPI reference run. The field coefficients were mapped on to the reference EPI using the transform. The BOLD reference was then co-registered to the T1w reference using bbregister (FreeSurfer) which implements boundary-based registration (Greve & Fischl, 2009). Co-registration was configured with six degrees of freedom. Several confounding time-series were calculated based on the preprocessed BOLD: framewise displacement (FD), DVARS and three region-wise global signals. FD was computed using two formulations following Power (absolute sum of relative motions (Power et al., 2014)) and Jenkinson (relative root mean square displacement between affines (Jenkinson et al., 2002)). FD and DVARS are calculated for each functional run, both using their implementations in Nipype (following the definitions by Power et al. (Power et al., 2014)). The three global signals are extracted within the CSF, the WM, and the whole-brain masks. Additionally, a set of physiological regressors were extracted to allow for component-based noise correction using CompCor (Behzadi et al., 2007). Principal components are estimated after high-pass filtering the preprocessed BOLD time-series (using a discrete cosine filter with 128s cut-off) for the two CompCor variants: temporal (tCompCor) and anatomical (aCompCor). tCompCor component are then calculated from the top 2% variable voxels within the brain mask. For aCompCor, three probabilistic masks (CSF, WM, and combined CSF+WM) are generated in anatomical space. The implementation differs from that of Behzadi et al. (Behzadi et al., 2007) in that instead of eroding the masks by 2 pixels on BOLD space, a mask of pixels that likely contain a volume fraction of GM is subtracted from the aCompCor masks. This mask is obtained by dilating a GM mask extracted from the FreeSurfer’s aseg segmentation, and it ensures components are not extracted from voxels containing a minimal fraction of GM. Finally, these masks are resampled into BOLD space and binarised by thresholding at 0.99 (as in the original implementation). Components are also calculated separately within the WM and CSF masks. For each CompCor decomposition, the k components with the largest singular values are retained, such that the retained components’ time series are sufficient to explain 50 percent of variance across the nuisance mask (CSF, WM, combined, or temporal). The remaining components are dropped from consideration. The head-motion estimates calculated in the correction step were also placed within the corresponding confounds file. The confound time series derived from head motion estimates and global signals were expanded with the inclusion of temporal derivatives and quadratic terms for each (Satterthwaite et al., 2013). Frames that exceeded a threshold of 0.5mm FD or 1.5 standardised DVARS were annotated as motion outliers. Additional nuisance timeseries are calculated by means of principal components analysis of the signal found within a thin band (crown) of voxels around the edge of the brain, as proposed by Patriat, Reynolds, and Birn (Patriat et al., 2017). The BOLD timeseries were resampled onto the left/right-symmetric template “fsLR” using the Connectome Workbench (Glasser et al., 2013). Grayordinates files (Glasser et al., 2013) containing 91k samples were also generated with surface data transformed directly to fsLR space and subcortical data transformed to 2mm resolution MNI152NLin6Asym space. All resamplings can be performed with a single interpolation step by composing all the pertinent transformations (i.e. head-motion transform matrices, susceptibility distortion correction when available, and co-registrations to anatomical and output spaces). Gridded (volumetric) resamplings were performed using nitransforms, configured with cubic B-spline interpolation. Many internal operations of fMRIPrep use Nilearn 0.10.4 (Abraham et al., 2014; *SciCrunch | Research Resource Resolver*, n.d.-g), mostly within the functional processing workflow. For more details of the pipeline, see the section corresponding to workflows in fMRIPrep’s documentation.

**A6 Thermometer Level Data.**


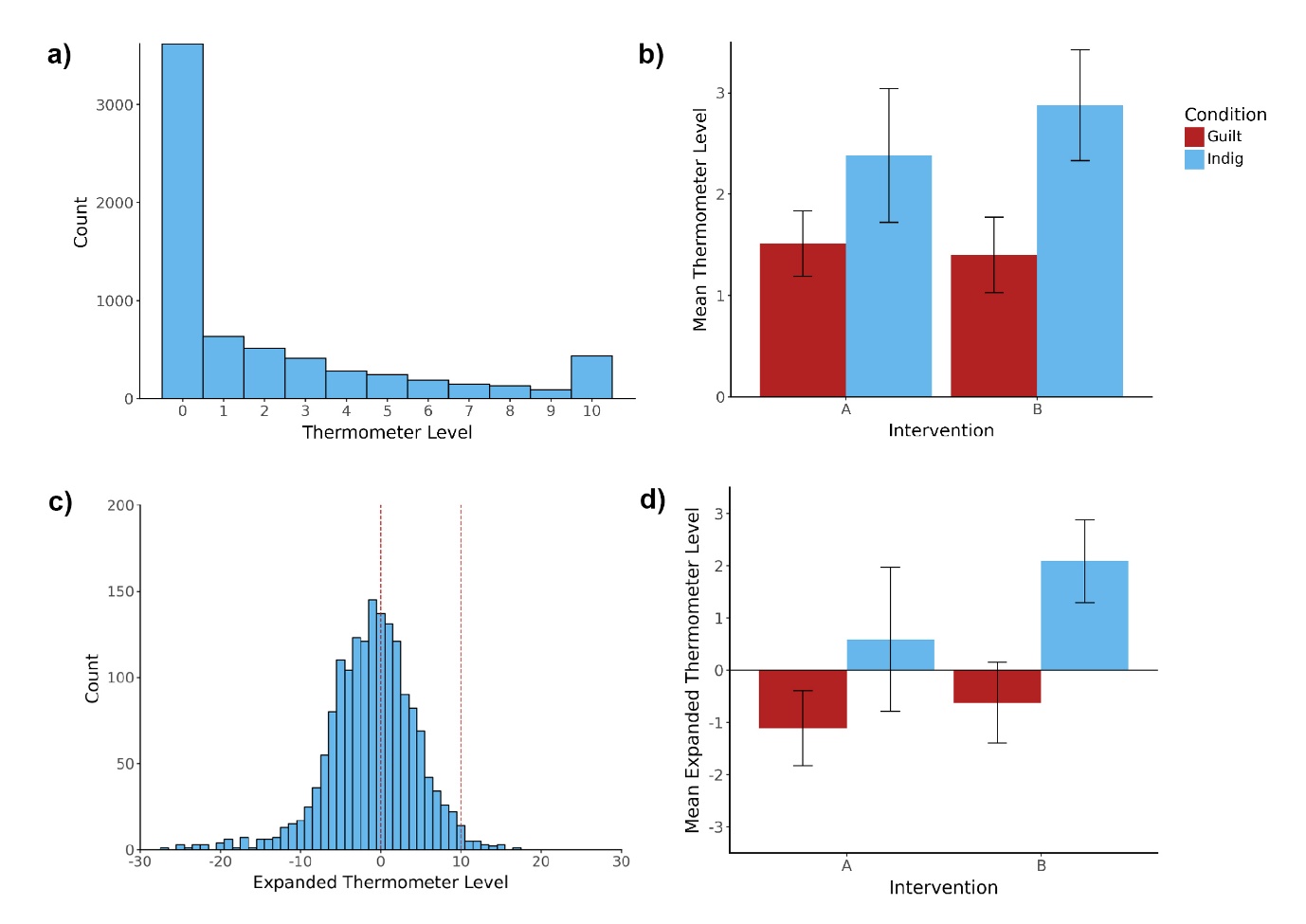


Figure 4. Thermometer level data. ***a)*** *Histogram of the entire 0-10 thermometer dataset, ungrouped by participant, nor condition or intervention. Floor and ceiling effects can be seen here.* ***b)*** *Plot of mean 0-10 thermometer levels (*$\pm$*SE), grouped by participant and then by factor combination. The mean levels for indignation are higher in each intervention group compared to guilt.* ***c)*** *Histogram of the expanded thermometer level data for guilt in Intervention A. The data was not first pooled by participant. The vertical red dashed lines in each plot illustrate the 0-10 level range.* ***d)*** *Mean expanded thermometer levels (*$\pm$*RSE) for each factor combination, grouped first by participant and then by factor combination.*

A7 Thermometer and Clinical Data Correlations.

Table 2. Correlations of expanded thermometer levels with clinical assessment score changes for each factor combination between Visit 1 and Visit 3. *Positive correlations for the Rosenberg self-esteem assessment and PANAS positive affect scale (PANAS+) represent clinical improvements. For the other clinical assessments, negative correlations represent clinical improvements. Correlation strength is measured by the Pearson correlation coefficient (r) and associated p-value (p). P-values reported are uncorrected. When applying Benjamini-Hochberg FDR correction for the 24 comparisons, no correlations reached statistical significance (p < 0.05).*

|  | | Guilt  + Int. A | Indignation + Int. A | Guilt  + Int. B | Indignation + Int. B |
| --- | --- | --- | --- | --- | --- |
| Rosenberg | **r** | 0.07 | 0.60 | -0.06 | 0.30 |
|  | **p** | 0.84 | 0.07 | 0.89 | 0.41 |
| MADRS | **r** | -0.49 | -0.26 | -0.36 | -0.49 |
|  | **p** | 0.15 | 0.47 | 0.31 | 0.16 |
| QIDS | **r** | -0.07 | -0.20 | 0.10 | -0.31 |
|  | **p** | 0.84 | 0.58 | 0.77 | 0.39 |
| GAD | **r** | 0.77 | -0.22 | 0.11 | 0.10 |
|  | **p** | 0.01 | 0.53 | 0.77 | 0.79 |
| PANAS+ | **r** | 0.54 | 0.03 | -0.08 | 0.45 |
|  | **p** | 0.10 | 0.93 | 0.84 | 0.19 |
| PANAS- | **r** | 0.42 | -0.12 | -0.28 | 0.29 |
|  | **p** | 0.23 | 0.73 | 0.43 | 0.41 |

**A8 Individual Participant Clinical Assessment Data.**


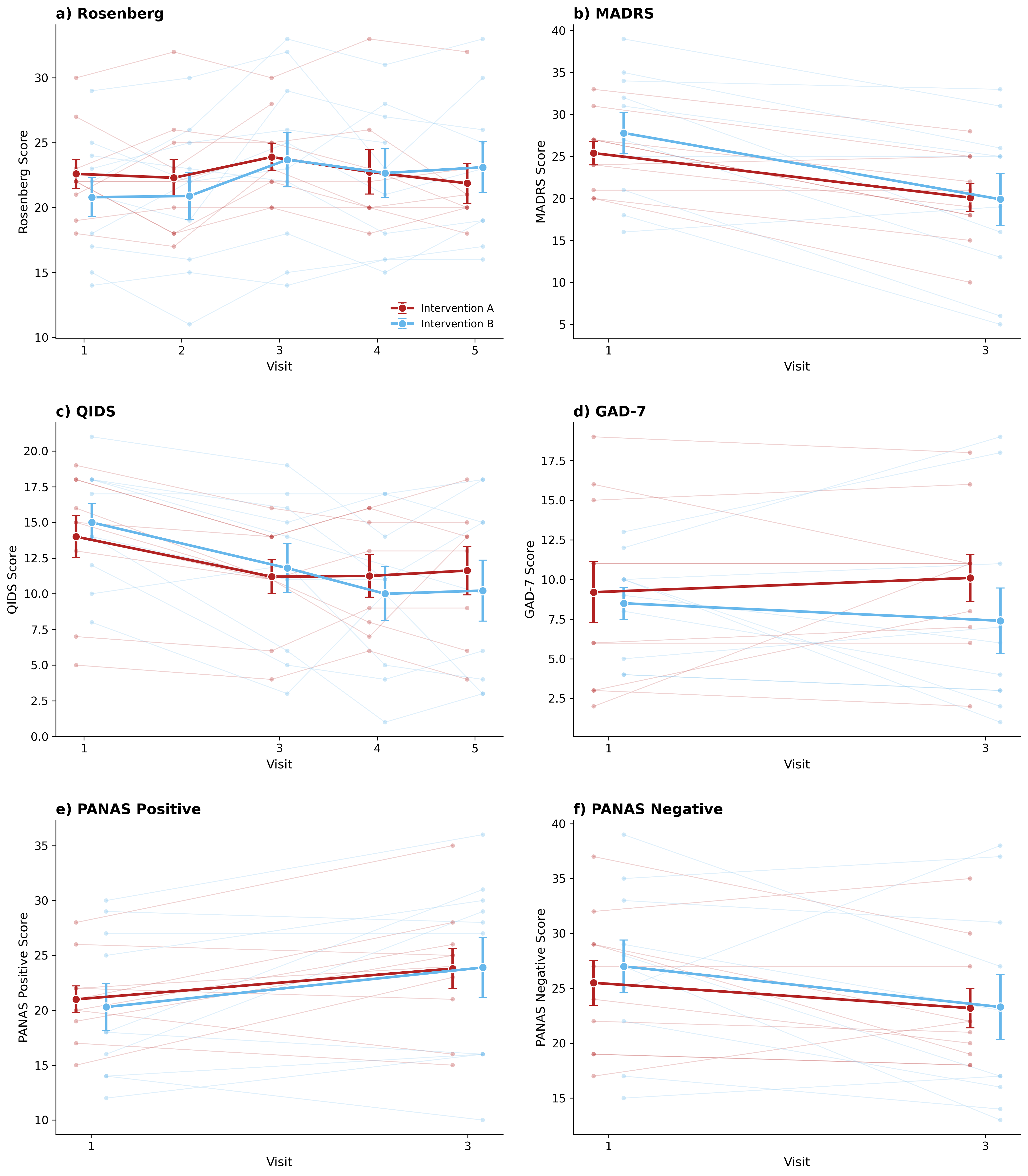


***Figure 5. Clinical assessment scores with individual participant trajectories.*** *Each panel displays group mean scores (±SE; thick lines with error bars) for Intervention A (red) and Intervention B (blue), with individual participant trajectories overlaid as thin, semi-transparent lines. a) Rosenberg self-esteem scores (Visits 1–5). b) MADRS depression scores (Visits 1 and 3). c) QIDS depression scores (Visits 1, 3, 4, and 5). d) GAD-7 anxiety scores (Visits 1 and 3). e) PANAS positive affect scores (Visits 1 and 3). f) PANAS negative affect scores (Visits 1 and 3).*

**A9 Individual Participant Thermometer Data.**

***Table 3. Results of one-sample t-tests, against 0, of expanded thermometer levels for each condition for each participant.*** *P-values were adjusted using the Bonferroni method to correct for 40 comparisons. The ‘Therm. Direction’ column indicates the direction of significant thermometer movement based on adjusted p-values; a dash indicates the result was not significant after correction. (*p<0.05, **p<0.01, ***p<0.001).*

| **Participant** | **Intervention** | **Condition** | **Test Type** | **P-Value** | **Significance Level** | **Adjusted P-Value** | **Adjusted Significance Level** | **Therm. Direction** |
| --- | --- | --- | --- | --- | --- | --- | --- | --- |
| P004 | A | Guilt | Non-parametric | <0.001 | *** | <0.001 | *** | Decrease |
|  |  | Indignation | Non-parametric | <0.001 | *** | <0.001 | *** | Increase |
| P006 | A | Guilt | Non-parametric | 0.003 | ** | 0.108 | - | - |
|  |  | Indignation | Non-parametric | <0.001 | *** | <0.001 | *** | Increase |
| P020 | B | Guilt | Non-parametric | <0.001 | *** | <0.001 | *** | Decrease |
|  |  | Indignation | Non-parametric | 0.442 | - | 1.000 | - | - |
| P030 | B | Guilt | Non-parametric | 0.549 | - | 1.000 | - | - |
|  |  | Indignation | Non-parametric | <0.001 | *** | 0.002 | ** | Increase |
| P059 | B | Guilt | Parametric | 0.001 | ** | 0.053 | - | - |
|  |  | Indignation | Parametric | <0.001 | *** | <0.001 | *** | Increase |
| P078 | B | Guilt | Non-parametric | <0.001 | *** | <0.001 | *** | Decrease |
|  |  | Indignation | Non-parametric | <0.001 | *** | <0.001 | *** | Increase |
| P093 | B | Guilt | Non-parametric | <0.001 | *** | <0.001 | *** | Decrease |
|  |  | Indignation | Non-parametric | <0.001 | *** | <0.001 | *** | Increase |
| P094 | B | Guilt | Non-parametric | 0.096 | - | 1.000 | - | - |
|  |  | Indignation | Non-parametric | <0.001 | *** | <0.001 | *** | Decrease |
| P100 | A | Guilt | Parametric | <0.001 | *** | <0.001 | *** | Decrease |
|  |  | Indignation | Parametric | <0.001 | *** | <0.001 | *** | Decrease |
| P107 | B | Guilt | Non-parametric | 0.094 | - | 1.000 | - | - |
|  |  | Indignation | Non-parametric | <0.001 | *** | <0.001 | *** | Increase |
| P122 | A | Guilt | Non-parametric | 0.121 | - | 1.000 | - | - |
|  |  | Indignation | Non-parametric | <0.001 | *** | <0.001 | *** | Decrease |
| P125 | A | Guilt | Parametric | 0.014 | * | 0.572 | - | - |
|  |  | Indignation | Parametric | <0.001 | *** | <0.001 | *** | Increase |
| P127 | B | Guilt | Non-parametric | <0.001 | *** | 0.033 | * | Decrease |
|  |  | Indignation | Non-parametric | <0.001 | *** | <0.001 | *** | Increase |
| P128 | A | Guilt | Non-parametric | <0.001 | *** | <0.001 | *** | Decrease |
|  |  | Indignation | Non-parametric | <0.001 | *** | <0.001 | *** | Decrease |
| P136 | A | Guilt | Parametric | <0.001 | *** | <0.001 | *** | Increase |
|  |  | Indignation | Parametric | <0.001 | *** | <0.001 | *** | Decrease |
| P145 | A | Guilt | Non-parametric | <0.001 | *** | <0.001 | *** | Increase |
|  |  | Indignation | Parametric | 0.003 | ** | 0.137 | - | - |
| P155 | B | Guilt | Parametric | 0.001 | ** | 0.053 | - | - |
|  |  | Indignation | Parametric | <0.001 | *** | <0.001 | *** | Increase |
| P199 | B | Guilt | Non-parametric | <0.001 | *** | 0.009 | ** | Increase |
|  |  | Indignation | Non-parametric | <0.001 | *** | <0.001 | *** | Decrease |
| P215 | A | Guilt | Non-parametric | <0.001 | *** | <0.001 | *** | Decrease |
|  |  | Indignation | Non-parametric | <0.001 | *** | <0.001 | *** | Increase |
| P216 | A | Guilt | Non-parametric | <0.001 | *** | <0.001 | *** | Decrease |
|  |  | Indignation | Parametric | 0.004 | ** | 0.154 | - | - |

**A10 Individual Participant Thermometer Level Data.**


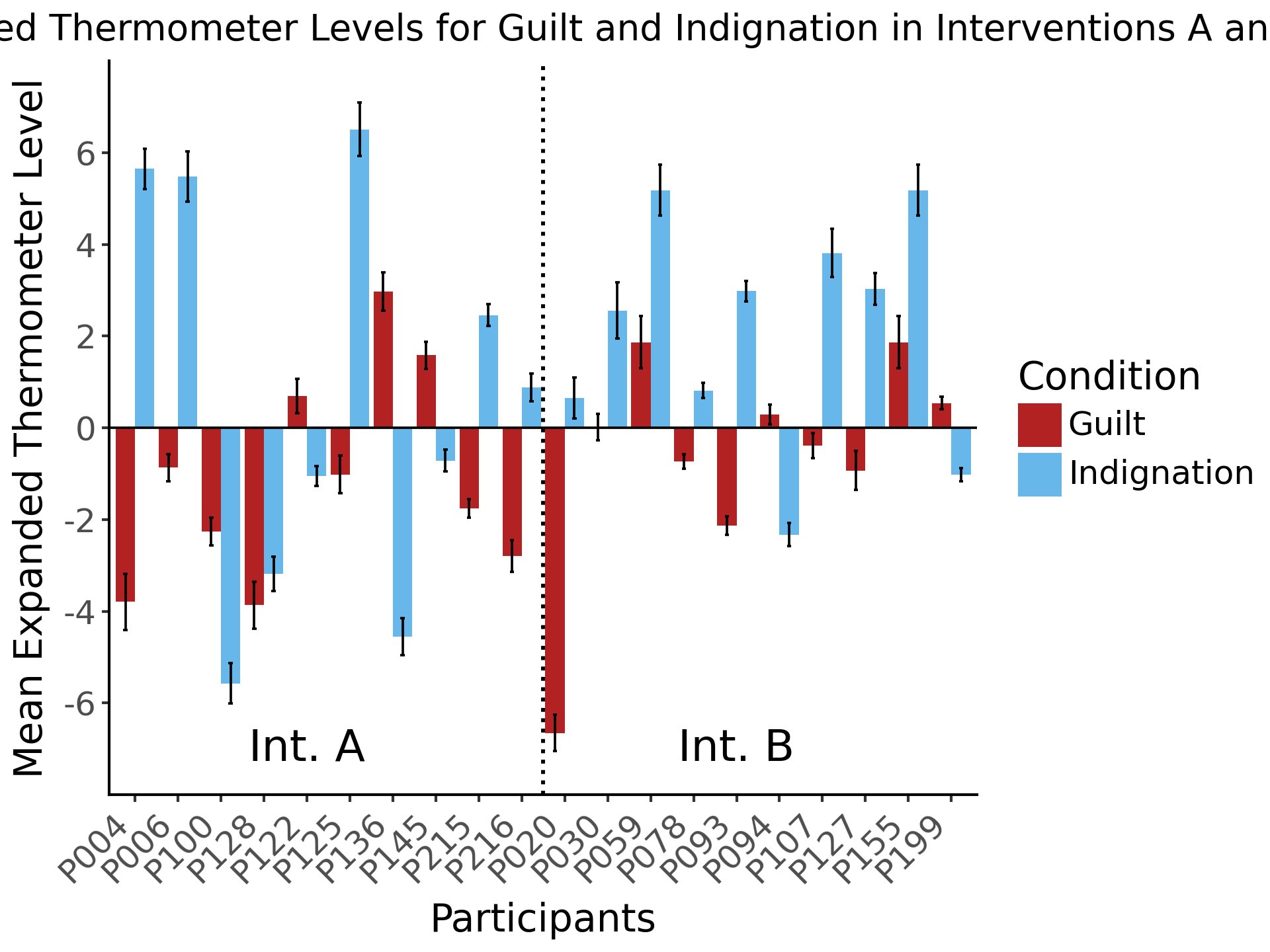


***Figure 6. Mean expanded thermometer levels (±SE) for each condition for each individual participant.*** *Participants from Intervention A are displayed in the first half of the x-axis (left of the vertical dotted line) and participants from Intervention B in the second half. Red bars represent the guilt condition and blue bars represent the indignation condition. Higher thermometer levels represent SCC upregulation in the guilt condition of Intervention A and the indignation condition of Intervention B; conversely, higher thermometer levels represent SCC downregulation in the indignation condition of Intervention A and the guilt condition of Intervention B. Error bars denote standard error of the mean.*

**A11 Primary Whole-Brain Analysis: Uniform (No-Fieldmap) Pipeline.**

The primary whole-brain analysis reported in the main text was conducted without applying fieldmap-based distortion correction to any participant (N = 20), ensuring a fully uniform preprocessing pipeline and avoiding the introduction of pipeline heterogeneity into the group-level spatial analysis. Data were processed using fMRIPrep 24.0.1 (MNI152NLin6Asym space, 2 mm resolution) and analysed using a three-level GLM in FEAT (FSL 6.0) with FLAME 1+2 mixed effects modelling at the group level (df = 18), in the same way as described in A5 of the Supplementary Materials (but without fieldmap application). Estimated smoothness was 7.2 × 7.1 × 6.8 mm FWHM. The group-level mask covered 130,044 voxels across the intersection of all participants, reflecting signal dropout in susceptibility-affected regions.

For the between-group contrast (Intervention B > Intervention A for the indignation > guilt, post > pre-neurofeedback contrast), no clusters survived whole-brain cluster-corrected thresholding (z > 2.3, cluster significance p < 0.05). The strongest, sub-threshold, signal was located in the left temporoparietal region (peak MNI coordinates −58, 6, −6; z-max = 3.64). Because no clusters survived correction, no thresholded statistical map is shown. This uniform-pipeline analysis constitutes the primary neuroimaging result and indicates no statistically significant between-group difference in the change in task-related activation at the whole-brain level in this small sample.

**A12 Exploratory Whole-Brain Analysis: GLM with Fieldmap Correction Where Available.**

For completeness and transparency, an exploratory analysis was conducted in which fieldmap-based distortion correction was retained for the 13 participants for whom fieldmaps were available, while the remaining 7 participants (who lacked fieldmaps owing to an acquisition error) were modelled with an additional regressor at the third level. Under this mixed pipeline, two left-lateralised clusters survived whole-brain cluster-corrected thresholding (cluster-forming threshold z > 2.3; cluster significance p < 0.05, corrected) for the indignation > guilt, post > pre-neurofeedback, Intervention B > Intervention A contrast (Table 4; Figure 7). Because this analysis combines images preprocessed with and without susceptibility distortion correction within a single group-level spatial analysis, it is vulnerable to alignment confounds in susceptibility-prone regions, and these clusters did not reproduce under the uniform primary pipeline (A11). This result is therefore reported as exploratory and interpreted with caution.

***Table 4. Clusters returned under the exploratory mixed-pipeline GLM for the ‘indignation > guilt and Run 4 > Run 1 and Intervention B > Intervention A’ contrast.*** *For each cluster, the top 3 most likely regions that the cluster corresponds to are given, based on the Harvard-Oxford Cortical and Subcortical Structural Atlases. Voxel counts refer to the number of voxels within each cluster surviving the statistical thresholding procedure (cluster-forming threshold z > 2.3; cluster significance p < 0.05, corrected).*

| **Cluster** | **Cluster Region(s)** | **Hemisphere** | **z-max** | **z-max MNI Coordinates** | **Number of Voxels** | **p-value** | **PSC (%)** |
| --- | --- | --- | --- | --- | --- | --- | --- |
| 1 | Parietal operculum cortex  Planum temporale  Left cerebral white matter | Left | 3.89 | x=−56.5, y=−24.5, z=17.5 | 959 | <0.001 | 1.94 |
| 2 | Superior lateral occipital cortex  Occipital pole  Left cerebral white matter | Left | 3.67 | x=−26.5, y=−88.5, z=31.5 | 402 | 0.010 | 1.74 |


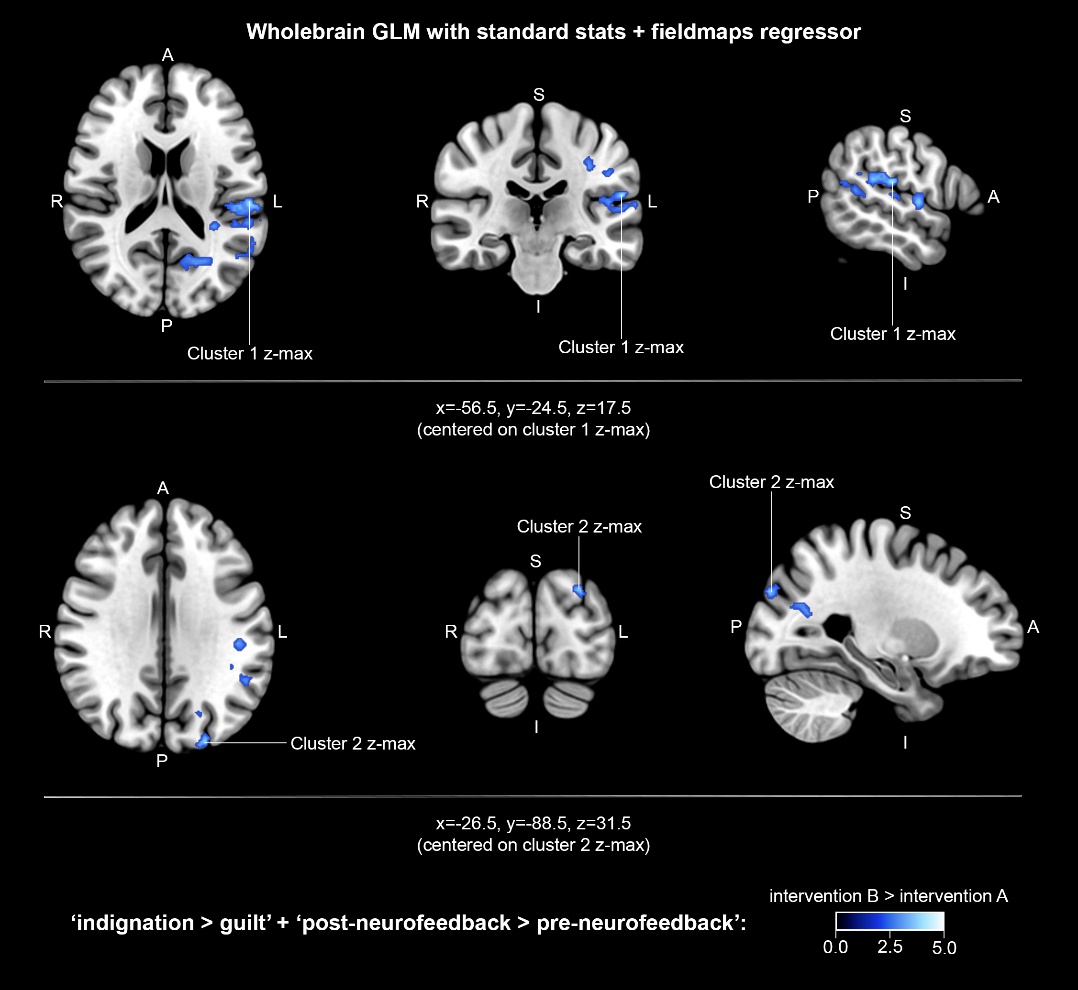


***Figure 7. Z-statistic brain map from the exploratory mixed-pipeline whole-brain GLM showing two clusters of activation.*** *Brain maps showing z-statistic values (colour bar) for voxels within clusters surviving cluster-based thresholding (cluster-forming threshold z > 2.3; cluster significance p < 0.05, corrected) in the exploratory analysis that retained fieldmap correction where available and modelled its absence with an additional third-level regressor. The first row contains a transverse, coronal, and sagittal slice centred on the first cluster’s max z-stat value (MNI coordinates: x=−56.5, y=−24.5, z=17.5). The second row is centred on the second cluster’s max z-stat value (MNI coordinates: x=−26.5, y=−88.5, z=31.5). These clusters were identified for the indignation > guilt contrast, from Run 1 to Run 4 (pre- to post-neurofeedback), in Intervention B participants relative to Intervention A. The colour bar ranges from 0 to 5; only voxels within clusters surviving the statistical threshold are displayed, but the colour bar begins at 0 to optimise visualisation of supra-threshold voxels. As noted above, these clusters did not survive the uniform primary pipeline (A11) and are reported as exploratory.*


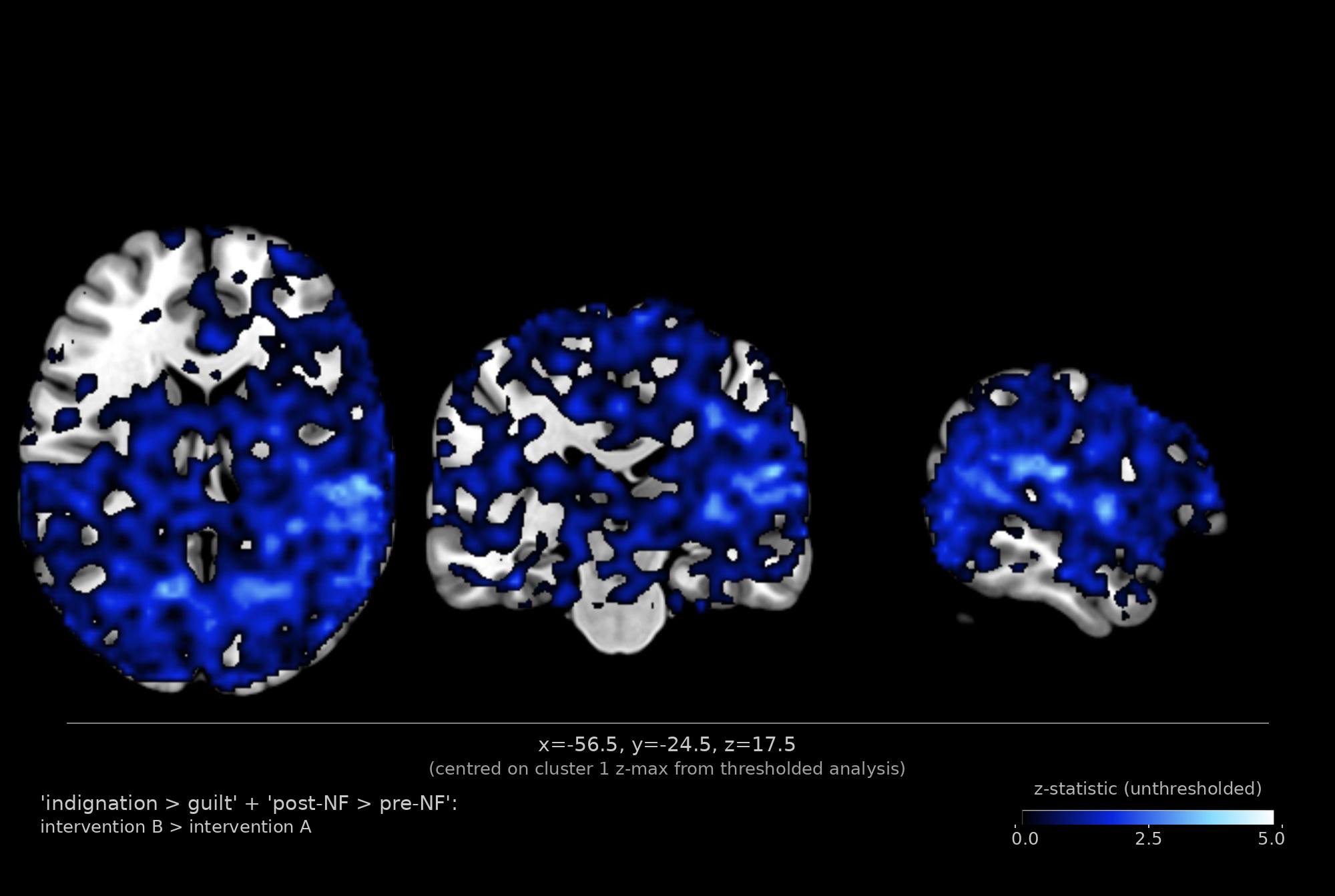


***Figure 8. Unthresholded z-statistic brain map from the exploratory mixed-pipeline whole-brain GLM.*** *Transverse, coronal, and sagittal slices centred on the z-max of cluster 1 from the thresholded exploratory analysis (MNI coordinates: x=−56.5, y=−24.5, z=17.5). The colour bar represents unthresholded z-statistic values (range 0–5) for the indignation > guilt, post > pre-neurofeedback, Intervention B > Intervention A contrast, with an additional fieldmaps regressor included at the third level. This map is provided for transparency to allow readers to assess the spatial distribution of the underlying signal across the whole brain (Taylor et al., 2025). Compare with the thresholded version in Figure 7.*
